# Family Medicine as a Model of Primary Health Services Delivery: A Pilot Study in Almaty, Kazakhstan

**DOI:** 10.5195/cajgh.2015.209

**Published:** 2015-04-08

**Authors:** Dilara Orynbassarova

**Affiliations:** KIMEP University, MPA, Almaty, Kazakhstan

**Keywords:** Central Asia, Kazakhstan, primary health care, family medicine, access to care, general practice

## Abstract

**Introduction:**

Advanced models of delivering primary health care are being implemented in various countries of the world. This is especially true for countries undergoing a healthcare transition in Central Asia, such as Kazakhstan, which obtained independence from Soviet Union in 1991. The Kazakhstan National Program of Health Reform, implemented between 2005–2010, aimed to create an effective system of primary care. One of the key directions of healthcare reform implemented in Kazakhstan included the development of family medicine, which has become cutting-edge agenda for Kazakhstan Health Ministry over the past 10 years. While many papers have been published about the importance of family medicine and primary healthcare models, few have focused on analyzing family medicine effectiveness in Kazakhstan and its impact on access to family doctor services and patient satisfaction. The key aims of this pilot investigation were 1) to assess the model’s impact on access to primary care and patients’ satisfaction, and 2) to explore the model’s effectiveness in some Central Asian and transitional countries in the literature.

**Methods:**

This pilot study was based on semi-structured interviews and questionnaires about the perception and impact of the primary care model to 86 respondents aged 19–51 (54% females, 46% males). The majority of respondents were Almaty city residents (71%), while the rest were Almaty Province rural residents (22%) and residents of other Kazakhstan regions (7%).

**Results:**

Respondents from rural areas associated general practitioners, or family doctors, with community clinics (also referred to as feldsher posts). Even though urban area respondents use family doctor services, they were more likely to get those services in private rather than public clinics. Rural residents appear to have better access to primary care providers than urban residents participating in our study. Also, respondents from rural areas were more satisfied with services provided by family doctors than respondents from urban areas.

**Conclusions:**

This pilot study helped to improve our understanding of primary health care reforms implemented in Kazakhstan, a topic that is not traditionally covered in international literature. This pilot study suggests that primary care is more effectively implemented in rural areas of Kazakhstan (Almaty Province); however, future full-scale research in this area is needed to fully understand the complexity of primary healthcare access in Kazakhstan.

Advanced models of delivering primary health care are being implemented in various countries around the world. This is especially true for countries undergoing a healthcare transition in Central Asia, such as Kazakhstan, which obtained independence from Soviet Union in 1991. Many international declarations and forums recognized the importance of implementing an effective primary health care system.^1^ Primary health care is an effective vehicle to “improve health care access and outcomes while narrowing equity gap.”^2^ Qualitative analysis of empirical evidence indicates one of the directions that most countries undertake while reforming their primary care system is the development of family medicine centered care, which has become a critical component of Western public health systems. Published evidence indicates that 90% of various patients in different countries around the world begin and end their medical treatment at the level of a family doctor, seeking help of highly specialized professionals only in exceptional cases.^2^–^4^

As a country in transition, Kazakhstan is seeking the most appropriate model for improving public health practice, traditionally challenged by a focus on a curative medicine approach rather than prevention.^5^–^7^ Despite the fact that several drawbacks of primary healthcare models have been recognized, including access to care, various publications recognized positive aspects of primary care models, including slower growth in healthcare spending.^8^ Recent publications by Sharman,^9^–^11^ emphasized the need for change from Kazakhstan’s current disease-centric healthcare paradigm to a new primary health and wellness-centric health care paradigm.^11^ The concept of primary care is not unknown in Kazakhstan, as historically, in the countries of the former Soviet Union, many functions of primary care doctors have been performed by feldshers.^12^ Feldsher is a middle level healthcare provider with training similar to physician assistants in the US or an advanced nursing degree, providing primary care mainly in rural areas in the countries of former Soviet Union. Also, while it had many weaknesses, Semashko’s centralized model of healthcare, as was practiced during the Soviet period, paid particular attention to prevention, especially in the area of vaccinations.^13^

One of the major objectives of the Kazakhstani Health Program 2005–2010 and part of 2020 strategic development plan, included the development of family medicine and strengthening the principles of general medical practice. As a part of this program’s vision, family doctors would gradually replace current healthcare providers, such as district physicians and pediatricians, thus unifying the primary care services delivery system.^14^,^15^ Family medicine-centered care in Kazakhstan is not new health care developmenal approach; it was introduced in Kazakhstan over twenty years ago. The history of introducing the family medicine model started in 1990s, when the first pilot clinic of mixed type (district physicians practicing with family doctors) was created in Alma Ata (Almaty) in 1989.^16^–^18^ Little has been published about assessing family medicine practice implications for Kazakhstan, and almost no research has been conducted about the impact of the model on access to primary care services and satisfaction with those services by Kazakhstani citizens. The main aim of this pilot investigation was to investigate the care given under the family medicine model in Kazakhstan, and observe if any disparity between rural and urban settings exist regarding access to care and satisfaction with these services.

Results of this pilot study can serve as a foundation for further full-scale research studies in the fields of primary care, family medicine, and general medical practice in Kazakhstan. This research would be of special interest to those with interest in Central Asian region.

## Methods

Survey development for this study was based on elements of Hsiao Evaluation Framework elements, including organizational arrangements, financing, resource allocation, and provision elements, which refers to allocation of health care goods and services.^19^,^20^ Since this pilot study focused on finding out the family medicine effectiveness in providing healthcare services, the service provision element was the key target for the analysis. Effective service provision indicators included access to primary care services, patient satisfaction, and capacity of effectively managing acute and chronic diseases by family doctors.

Data were collected from November 1, 2009-January 12, 2010. The main data collection technique was a survey tool composed of semi-structured interviews and questionnaires created for this study. The questionnaire was divided into five major parts as per effective service provision indicators. Interviews were conducted by two highly trained professionals, including a district pediatrician and district therapist. This pilot study consisted of 86 respondents aged 19–51 (54% females, 46% males). The majority of respondents were Almaty city residents (71%), while the rest were Almaty Province rural residents (22%), and residents of other Kazakhstan regions (7%). Descriptive statistics have been conducted to report the findings.

Convenience sampling technique was applied in a randomly chosen Almaty City Polyclinic #8 and Almaty Diagnostic Hospital. Interviews were conducted by specialists, who had approximately 7 years of experience working with the Association of Family Doctors of Kazakhstan, district pediatricians, who had roughly 15 years of experience working in the Almaty Child City Polyclinic #8, and district therapists, who had approximately 11 years of experience working in Almaty City Polyclinic #8 and worked part-time as a family doctor in the Republican Family-Doctor Center in Almaty. Patients who were available to complete the survey were surveyed.

While no formal ethics review was conducted, this project was approved as thesis work by KIMEP University. Each questionnaire also had a heading that informed participants of the aims of the study in addition to the confidentiality of their responses.

## Results

One of the key findings of this study was that rural area respondents associated feldshers with primary care doctors. It was observed that the majority of respondents (75%) from rural areas responded that the community feldsher doctor was their first contact, while 15% of respondents had difficulties in replying to the survey, and 10% of respondents cited other as their primary contact in the case of a health issue. Even though urban area patients used family medicine services, they preferred to get those services in private clinics vs. public ones. Specifically, 71% of urban respondents indicated a preference for private clinics, 26% indicated preference for public clinic services, and 3% found it difficult to respond. Respondents reported that the reasoning for this may be that quality doctors are not motivated to work in public clinics due to low financial incentives. Also, there is a lack of incentives for serving children, adults, and women all together by one family doctor, as district therapists are better reimbursed by serving mainly the adult population.

Despite the small sample size, it appears that rural residents have higher access to family medicine services compared to urban residents. Almost half of rural respondents (49%) agreed with the statement “patients can contact family doctor easily by telephone to obtain advice.” The majority of urban respondents (55%) disagreed with the statement “the appointments are easy to make whenever I need them” compared to 45% of rural patients agreeing with this statement. 55% of urban patients indicated that the public polyclinic they visited did not employ a family doctor.

Rural area respondents in this study were more satisfied with services provided by family doctors than respondents from urban areas. Overall, 51% of respondents were dissatisfied with the quality of services provided by family doctors, 2% very dissatisfied, 12% moderately satisfied, 31% satisfied, and 4% very satisfied*.* Groups of dissatisfied respondents (51%) and satisfied (31%) were targeted for selective analysis by their place of residence. Considerable difference in answers was revealed. Among 51% respondents dissatisfied by family doctor services, 82% were Almaty city residents, and among 31% respondents satisfied, 71% of respondents were from rural areas.

Based on qualitative interview data, it appears that patients living in rural communities in Kazakhstan are more satisfied with services provided by family doctors since they have less access to specialist care, and urban area patients prefer directly accessing specialists in secondary and tertiary care specialized hospitals, since those are available in Almaty city. Data analysis revealed that rural patients considered the family doctor as point of first contact for acute and some of the chronic health problems, while urban respondents considered the district therapist as doctor of first contact for acute health problems, and preferred narrow field specialists as a first contact doctor for chronic illnesses. The majority of urban respondents would rarely refer to family doctors when having chronic illness, including gastritis, allergy, chest pain, or ENT problems, and prefer to visit specialists for those. In allergy cases, the majority of urban respondents (52%) would refer to allergologyst, chest pain (63%) to cardiologist, applying plaster cast (66%) to surgeon, and vision problem 48% to ophthalmologist. However, different answers revealed from rural patients. The majority rural respondents would consider the family doctor as the doctor of first contact when having health problems and some chronic illnesses, such as gastritis and ENT problems.

## Discussion

In summary, the family medicine model is being practiced in Kazakhstan, and appears to be accessible to rural residents. An interesting finding is that even though urban area respondents used family doctor services, they preferred to get those services in private clinics. Countries in transition, including Armenia, Russia, Kyrgyzstan, and Turkmenistan that inherited the Soviet-Semashko model of health care, and characterized by a centrally controlled healthcare service provision, were found to have similarly developed levels of a family medicine model, with rural areas being more positively affected by the primary care model compared to urban ones. For comparison, the same trend is observed in the Kyrgyzstan study, where rural family doctors in Issyk Kul (rural area) managed the initial visit with health concerns 80–100% of the time more frequently than in Bishkek city (capital city).^21^ The presence of a large number of highly trained specialists in urban areas as an alternative to family doctors have adversely impacted family doctors’ ability to solve first-contact chronic health problems. A higher density of population in urban *vs*. rural areas, as well as a high concentration of specialists in urban areas may potentially prevent urban area family doctors to fully realize their potential.

Limitation of this research includes small sample size, poor representatives of rural population, and non-randomized nature of our sampling technique. While these weaknesses limited the generalizability of study findings to the general population of Kazakhstan, these limitations will be addressed in future research. However, since there is a paucity of published research on primary healthcare models in Kazakhstan, this pilot investigation has been designed to fill this important gap. To generate more nationally representative samples, further research is suggested that would capture a wider variety of geographic catchment areas, with random selection of research participants. Future studies should specifically address rural-urban differences in perception and use of primary care. Kazakhstan is a good country to implement this research, as it has undergone substantial economic and political transition in a relatively short time frame.

Family medicine model care was found to be effective and accessible, especially in rural areas of Kazakhstan. Better policies targeting primary care integration should be considered for furthering primary care in Kazakhstan. While it early to judge the overall effectiveness of the model, work to date clearly indicates that impacts and challenges of this model for Kazakhstan need to be assessed. Advantages and disadvantages of this model need to be clearly explored before further integration, as previous publication suggested that overall health expenditures were higher in countries with stronger primary care structures, perhaps because maintaining strong primary care structures is costly and promotes developments such as decentralization of services delivery.^8^

As an interesting comparison, studies in Kyrgyzstan, Armenia, and Turkmenistan suggested that family care models improved rural access of those residents with low income and low education.^22^–^24^ In Kyrgyzstan study, increased access to services positively impacted waiting time and out of pocket payments of (73%), as rural patients no longer needed to travel to urban centers for specialists’ care.^24^ Turkmenistan study indicated 2 fold decrease of Sayat Chardzhou rural total health expenditure and 3-fold decrease in patient hospitalization rate.^25^ In Moldova study, rural patients (68%) living in village near to Laloveni city expressed satisfaction with increased access to services.^26^ Ukrainian study of Dneprodzerzhinsk indicated a 3-fold decrease in ambulatory call frequency, and patient hospitalizations became lower than average city level.^27^ In Armenia, the proportion of self-referrals by patients to specialist care in rural areas decreased from 26% to 20%, in contrast, self-referrals rate in urban areas increased 22% to 38%.^24^

This pilot study helped to improve our understanding of primary health care reforms implemented in Kazakhstan, a topic that is not traditionally covered in international literature. This pilot study seems to suggest that primary care is more effectively implemented in rural areas of Kazakhstan (Almaty Province); however, future full-scale research in this area is needed to fully understand the complexity of primary healthcare access in Kazakhstan.
